# Letter to the editor in response “Commissioning of a near real‐time in vivo film dosimetry system”

**DOI:** 10.1002/acm2.70847

**Published:** 2026-10-14

**Authors:** Agnieszka Walewska, Paulina Wesołowska, Paweł Kukołowicz

**Affiliations:** ^1^ Department of Medical Physics The Maria Sklodowska‐Curie National Research Institute of Oncology Warsaw Poland

1

Dear Editor,

We read the article by Yunjie Yang et al., “Commissioning of near real‐time in vivo dosimetry,” with great interest.^1^ The Pnt‐Dos™ solution's particular value lies in its ability to significantly reduce the time between taking a measurement and obtaining a result. This is a major advantage of the Pnt‐Dos™ in vivo dosimetry system. However, in Yang's paper, we found no information on how the reference dose is determined, that is the dose against which the in vivo measurement result is compared. When measurements are taken for patients who receive the full prescribed dose over their entire body—for example, in total body irradiation techniques or electron therapy—the reference dose is the prescribed dose itself. However, in the case of other radiotherapy techniques, particularly IMRT and VMAT, the dose at the surface differs significantly from the prescribed dose. We encountered this issue when preparing our film detector for in vivo measurements.^2^ We resolved this issue by developing software that uses data retrieved from the treatment planning system (DICOM RT) to extract the dose from a depth of 3 mm, which is where the film is located within our detector. Tests carried out with our detector have shown that the software enables the precise determination of the reference dose for dynamic techniques. For the seven plans prepared using rotational techniques, the average discrepancy between the measured and calculated values was 1.4%, with a maximum discrepancy of 3.6%. We therefore adapted our build‐cup cover for the Pnt‐Dose™ system detectors, 3D‐printed it, and repeated the previous measurements for our detector. The results confirmed the methodology's and software's suitability for in vivo dose measurement using Pnt‐Dose™ on the body surface for dynamic techniques. We therefore adapted our build‐up cover for the Pnt‐Dose™ system detectors, 3D‐printed it, and repeated the previous measurements carried out for our detector. The results confirmed the suitability of the methodology and software for in vivo dose measurement using the Pnt‐Dose™ system on the body surface for dynamic techniques. For four VMAT plans, the average discrepancy between the measured and calculated values was 3.8%, with a maximum discrepancy of 6.3%. Integrating our proposed solution into the Pnt‐Dose™ system would significantly expand its range of applications without compromising accuracy.
